# Circadian variation in pulmonary inflammatory responses is independent of rhythmic glucocorticoid signaling in airway epithelial cells

**DOI:** 10.1096/fj.201800026RR

**Published:** 2018-07-02

**Authors:** Louise M. Ince, Zhenguang Zhang, Stephen Beesley, Ryan M. Vonslow, Ben R. Saer, Laura C. Matthews, Nicola Begley, Julie E. Gibbs, David W. Ray, Andrew S. I. Loudon

**Affiliations:** Division of Diabetes, Endocrinology, and Gastroenterology, School of Medical Sciences, University of Manchester, Manchester, United Kingdom

**Keywords:** circadian rhythms, inflammation, endocrinology, *Ccsp-GR^−/−^*, *LysM-GR^−/−^*

## Abstract

The circadian clock is a critical regulator of immune function. We recently highlighted a role for the circadian clock in a mouse model of pulmonary inflammation. The epithelial clock protein Bmal1 was required to regulate neutrophil recruitment in response to inflammatory challenge. Bmal1 regulated glucocorticoid receptor (GR) recruitment to the neutrophil chemokine, CXC chemokine ligand 5 (CXCL5), providing a candidate mechanism. We now show that clock control of pulmonary neutrophilia persists without rhythmic glucocorticoid availability. Epithelial GR-null mice had elevated expression of proinflammatory chemokines in the lung under homeostatic conditions. However, deletion of GR in the bronchial epithelium blocked rhythmic CXCL5 production, identifying GR as required to confer circadian control to CXCL5. Surprisingly, rhythmic pulmonary neutrophilia persisted, despite nonrhythmic CXCL5 responses, indicating additional circadian control mechanisms. Deletion of GR in myeloid cells alone did not prevent circadian variation in pulmonary neutrophilia and showed reduced neutrophilic inflammation in response to dexamethasone treatment. These new data show GR is required to confer circadian control to some inflammatory chemokines, but that this alone is insufficient to prevent circadian control of neutrophilic inflammation in response to inhaled LPS, with additional control mechanisms arising in the myeloid cell lineage.—Ince, L. M., Zhang, Z., Beesley, S., Vonslow, R. M., Saer, B. R., Matthews, L. C., Begley, N., Gibbs, J. E., Ray, D. W., Loudon, A. S. I. Circadian variation in pulmonary inflammatory responses is independent of rhythmic glucocorticoid signaling in airway epithelial cells.

Glucocorticoids (Gcs) are potent immune suppressors. They regulate cytokine and chemokine expression during inflammation, leading to decreased leukocyte infiltration to affected tissues (1–4). Endogenous Gcs are secreted in a highly rhythmic manner (5) with pulses every 1–2 h (an ultradian rhythm) and a major oscillation period of ∼24 h (a circadian rhythm). This rhythmic signal can also entrain rhythms in peripheral tissues, synchronizing the molecular clock present in cells throughout the body (6). Rhythmic Gc signaling may be integral to transcriptional oscillations in peripheral tissues and has been shown to regulate a large proportion of the liver transcriptome (7). However, very few studies have directly examined the importance of rhythmic Gc signals for coordinating rhythmic physiologic processes.

It is known that the Gc receptor (GR, encoded by *Nr3c1*) can directly interact with core clock components CRY1 and CRY2 to modulate GR-mediated transactivation, but that does not interfere with the repressive effects of Gc treatment upon inflammatory NF-κB target genes (8, 9). In contrast, Gc effects on inflammatory gene expression can be strongly modulated by the clock component REV-ERBα (*Nr1d1*) because REV-ERBα presence decreases the stability of GR and leads to an inverse relationship of nuclear localization between the 2 proteins (10). REV-ERBα may, therefore, be a candidate gatekeeper of GR nuclear entry and, thus, regulate GR function according to time of day. Furthermore, GR function may also be modified by posttranslational modifications of the receptor itself. GR possesses multiple sites for phosphorylation, acetylation, and SUMOylation, along with ubiquitination (reviewed in Kadmiel and Cidlowski 11). The BMAL1/CLOCK complex of the core clock itself can modify GR activity in this way, *via* CLOCK-mediated acetylation of the hinge region (12, 13).

The lung is acutely sensitive to both the immune-modulatory and time-keeping effects of Gc signaling, exhibiting oscillations in gene expression, which can be shifted by manipulating the concentrations of circulating Gcs over time (14). We have previously reported a strong time-of-day variation in pulmonary responsiveness to LPS, which depends on an intact circadian clock in airway epithelial cells (3). Rhythmic expression of the Gc-repressed gene *Cxcl5* and its subsequent secretion is a key epithelial cell signal regulating neutrophil influx from blood to lung. Disruption of Gc signaling *via* adrenalectomy (removing the endogenous ligand) blunts these oscillations, indicating a role for Gcs in driving the rhythmic phenotype observed. We, therefore, proposed that rhythmic Gc signaling in airway epithelial cells is critical for generating rhythms in pulmonary LPS response. This was tested by manipulating Gc signaling pharmacologically (*via* corticosterone clamp) and genetically in key target cells (airway epithelium and macrophages).

## MATERIALS AND METHODS

### Mice

Airway epithelium-specific GR-knockout mice were generated by crossing mice expressing a codon-improved Cre recombinase driven by the *Scgb1a1* [which encodes the club cell secretory protein (CCSP)] promoter (15), referred to herein as *Ccsp^iCre/+^*, with GR-floxed mice (B6.129P2-Nr3c1tm2Gsc/Ieg (16), henceforth referred to as *GR^flox/flox^*, obtained from the European Mutant Mouse Archive; Infrafrontier Research Infrastructure, Munich, Germany). Integration of the Cre sequence was examined by PCR on genomic DNA (forward primer 5′‑AGATGCCAGGACATCAGGAACCTG‑3′; reverse primer 5′‑ATCAGCCACACCAGACACAGAGATC‑3′). Macrophage-specific GR knockout mice were generated by crossing *LysM^Cre/+^* mice (17) with the *GR^flox/flox^* mice. Integration of the Cre sequence was examined by PCR on genomic DNA (forward primer: 5′‑CTTGGGCTGCCAGAATTTCTC‑3′, reverse primer: 5′‑CCCAGAAATGCCAGATTACG‑3′). All mice were on C57BL/6J background and were subsequently crossed with mice on an *mPer2^Luc^* background to facilitate bioluminescent imaging studies (18). Thus, the genetically modified strains used were *GR^flox/flox^mPer2LucCcsp^icre^*, henceforth known as *Ccsp-GR^−/−^* (epithelial GR knockout), *GR^flox/flox^mPer2LucLysM^cre^*, henceforth known as *LysM-GR^−/−^* (myeloid GR knockout), and the common littermate controls *GR^flox/flox^mPer2Luc*, henceforth known as *GR^WT^* (GR wild type).

Mice were housed in 12-h light/dark cycles with food and water *ad libitum*. Additional changes to lighting schedules are detailed in Supplemental Fig. 1. All experiments were performed in accordance with the Animals (Scientific Procedures) Act of 1986 (United Kingdom). In each experiment, we used age-matched, adult, male mice to minimize variability; specific details are given in figure legends and individual methods.

### Corticosterone clamp

C57BL/6J mice were bought from Envigo (Huntingdon, United Kingdom) at 8 wk on arrival and allowed to settle for 1 wk before surgery. All surgery was completed under isoflurane anesthesia. After shaving the nape of the neck, a small incision was made in the skin to form a pocket in which a pellet (21-d release corticosterone pellet; Innovative Research of America, Sarasota, FL, USA) was implanted. Pellets of 2.5, 5, or 7.5 mg (or vehicle control) were used to determine an appropriate dose, and 2.5 mg was used in experiments thereafter. The incision was sutured and mice were monitored for at least 1 wk before any further experiments. At the time of LPS challenge, mice were 11–12 wk old.

### Corticosterone ELISA

To measure circulating levels of corticosterone, blood samples were collected *via* tail tipping at zeitgeber time (ZT) 0 (lights on), ZT6 (midday), ZT12 (lights off), and ZT18 (midnight). Serum corticosterone levels were measured by an ELISA Kit, according to the manufacturer’s instructions (ADI-900-097; Enzo Life Sciences Inc., Farmingdale, NY, USA). Briefly, a 5-μl sample was mixed with an equal volume of steroid-displacement reagent. After 5 min of incubation, 190 μl of elution buffer was added to the mixture; 100 µl of the diluted samples were added to a 96-well plate, and standard protocols were then followed. Plates were read at 405 nm on a plate reader (BioTek Instruments, Winooski, VT, USA). Sample concentration was calculated from a standard curve fit to a semilog standard plot in Excel (Microsoft, Redmond, WA, USA).

### Nebulized LPS treatments

This procedure was performed as previously described by Gibbs *et al.* (3), in constant darkness to investigate the contribution of the endogenous pacemaker and avoid any potential masking or confounding effects of light. Briefly, after 24 h in constant darkness, mice (median age, 12 wk) were exposed to nebulized LPS (2 mg/ml, isolated from *Escherichia coli* 0127:B8, L3129; MilliporeSigma, Burlington, MA, USA) for 20 min in an acrylic chamber. Mice were euthanized 5 h later with i.p. pentobarbital. Lungs were lavaged with 1 ml bronchoalveolar lavage (BAL) fluid (10 mM EDTA in PBS) from tracheal cannulae. After centrifugation at 500 *g* for 5 min, BAL supernatant was stored at −80°C for protein concentration and ELISA measurement, whereas cell pellets were resuspended for a total cell count and flow cytometry. In some experiments, dexamethasone (Dex; 1 mg/kg, D2519; MilliporeSigma) or saline vehicle was administered intraperitoneally 1 h before LPS challenge (Supplemental Fig. 1).

### BAL cell analysis

Cell counts were obtained with a NucleoCounter NC-250 (chemometec, Lillerød, Denmark) total cell and viability assays on A8 slides. For flow cytometry, after blocking with anti-CD16/32 antibody (1:100, clone 93, 14-0161-86; Thermo Fisher Scientific, Waltham, MA, USA), cells were stained with anti-CD45 pacific blue (1:100, clone 30-F11, MCD4528; Thermo Fisher Scientific), anti-CD11c APC (1:200, clone N418, 17-0114-82; Thermo Fisher Scientific), and anti-Ly6G Alexa488 (1:200, clone RB6-8C5, 53-5931-82; Thermo Fisher Scientific) antibodies. Acquisition was performed with an LSR II machine (BD Biosciences, Franklin Lakes, NJ, USA), with ≥5000 CD45^+^ cells collected/sample. FlowJo software v.10 (Tree Star, Ashland, OR, USA) was used for analysis. BAL macrophages and neutrophils were defined as CD45^+^CD11c^+^Ly6G^−^ and CD45^+^CD11c^−^Ly6G^+^, respectively, and their concentrations were calculated by multiplying the percentage observed by the total cell count.

### BAL protein concentrations

BAL total protein concentration was measured by a Bicinchoninic Acid (BCA) Protein Assay Kit (BCA1 and B9643; MilliporeSigma). ELISA measurements were performed with R&D Systems brand ELISA kits (CXCL5: DY443, TNF-α: DY410, and IL6: DY406; Bio-Techne, Minneapolis, MN, USA).

### GR *in situ* hybridization

Lungs were inflated with a 50% OCT/50% PBS solution to maintain structure, snap frozen on dry ice, and stored at −80°C before cryosectioning. Twelve-micrometer sections were cut using a CM3050 cryostat (Leica Biosystems, Wetzlar, Germany) treated with RNaseZap (Thermo Fisher Scientific) to minimize RNA degradation. GR *in situ* hybridization was performed using prGR9 plasmid (a kind gift from Professor Karen Chapman, University of Edinburgh, Edinburgh, United Kingdom), as previously described by Gibbs *et al.* (3). Relative intensity from several lung sections was quantified in ImageJ software (National Institutes of Health, Bethesda, MD, USA).

### Immunohistology protocols

Lungs from naive mice were inflated with freshly prepared 4% paraformaldehyde and fixed overnight at 4°C. Tissue samples were then processed and embedded in paraffin wax before cutting 5-μm sections *via* microtome. GR immunofluorescence staining was performed by routine staining procedures, including antigen retrieval in sodium citrate buffer. Primary antibody (rabbit anti-GR, M-20; Santa Cruz Biotechnology, Dallas, TX, USA) was used at 1:100 dilution in TNB buffer (TSA kit with tetramethylrhodamine; PerkinElmer, Waltham, MA, USA), followed by biotinylated goat anti-rabbit at 1:500, streptavidin–horseradish peroxidase at 1:200, and tyramide substrate at 1:50. Mounting medium contained DAPI as a counterstain (Vector Laboratories, Burlingame, CA, USA).

### Bioluminescence imaging and photomultiplier tube experiments

Precision-cut lung sections were prepared *via* vibratome (Integrasection 7550MM; Campden Instruments, Loughborough, United Kingdom) with a thickness of 275 μm (19). Sections were placed on cell culture inserts within 35-mm, glass-bottomed dishes containing 1 ml of luciferin recording medium, sealed by a glass coverslip. Photon count was recorded in 1 h time bins with an LV200 luminescence microscopy system (Olympus, Tokyo, Japan), as previously described by Gibbs *et al.* (3). Bronchiole and parenchyma regions were selected and analyzed with ImageJ software. Bioluminescence from macrophages was recorded by a photomultiplier tube system, as previously described by Gibbs *et al.* (20). Data was detrended with a 24-h moving average and plotted as relative bioluminescence (photons/min). Phase change was calculated as the timing of the fourth peak of treated samples relative to the fourth peak of untreated samples (19). To test responses to ligands of the Gc and mineralocorticoid receptor (MR), we used corticosterone (100 nM; MilliporeSigma), RU486 (mifepristone, 1 µM; MilliporeSigma), Dex (200 nM; MilliporeSigma), 2 synthetic nonsteroidal compounds [67 and 69 (21)], used at 10 nM, a kind gift from Dr. Stuart Farrow, (GlaxoSmithKline, Brentford, United Kingdom), and an agonist of the MR spironolactone (1 µM; MilliporeSigma).

### Laser-capture microdissection

Mouse distal bronchiolar epithelial cell microdissection was performed as previously described by Betsuyaku and Senior (22). Approximately 0.8 ml of 50% v/v of OCT/PBS solution was installed *via* tracheal cannula into each mouse before samples were frozen in OCT on dry ice and stored at −80°C. Samples were cut into 7-µm sections onto PEN membrane slides (11600288; Leica Microsystems, ‎Wetzlar‎, Germany) using a cryostat. After serial dehydration in ethanol solutions (50, 70, 90, 100, and 100%, 30 s each), slides were allowed to air-dry for ∼2 min before being dissected under an LMD6500 microdissection system (Leica Microsystems). Distal bronchiolar epithelium up to 200 nm from the opening to alveolar areas was collected.

### Time-serial airway epithelial cell harvest

*Ccsp-GR^−/−^* and *GR^WT^* mice (median age, 12 wk) were housed in constant darkness for 24 h before lung collection (every 4 h for 44 h, 1 mouse/genotype/time point). Frozen lungs were used for laser capture to harvest airway epithelial cell RNA, as described above. Then, core circadian clock genes were measured by quantitative PCR (qPCR).

### qPCR

For qPCR, proprietary assays were used for *Cxcl5* (Mm00436451_g1), *Cxcl15* (Mm00441263_m1), and *GR* (Mm00433832_m1) (all Applied Biosystems brand; Thermo Fisher Scientific). Circadian clock gene primers were as follows: *Bmal1* forward, 5′‑CCGTGCTAAGGATGGCTGTT‑3′, reverse 5′‑TCTGTGTATGGGTTGGTGGC‑3′ (SYBR green-based assay); *Nr1d1* forward 5′‑ACGACCCTGGACTCCAATAA‑3′, reverse 5′‑CCATTGGAGCTGTCACTGTAGA‑3′, universal probe 52; *Nr1d2* forward 5′‑ACAGAAATAGTTACCTGTGCAACACT‑3′, reverse 5′‑GACTTGCTCATAGGACACACCA‑3′, universal probe 88; *Per2* forward 5′‑TCCGAGTATATCGTGAAGAACG‑3′, reverse 5′‑CAGGATCTTCCCAGAAACCA‑3′, universal probe 5; *Dbp* forward 5′‑CTTTTGACCCTCGGAGACAC‑3′, reverse 5′‑CCGGCTCCAGTACTTCTCAT‑3′, universal probe 82; *Cry1* forward 5′‑ATCGTGCGCATTTCACATAC‑3′, reverse 5′‑TCCGCCATTGAGTTCTATGAT‑3′, universal probe 85 (all F. Hoffmann-La Roche, Basel, Switzerland). *18s* expression was used as an internal control (forward 5′‑CTCAACACGGGAAACCTCAC‑3′, reverse 5′‑CGCTCCACCAACTAAGAACG‑3′ and universal probe 77 (F. Hoffmann-La Roche). Samples were run in a StepOnePlus Real-Time PCR System machine (Thermo Fisher Scientific) and quantified by the cycle threshold (2^−ΔΔ^*^Ct^*) method.

### Nanostring

Nebulized LPS challenge was performed in *Ccsp-GR^−/−^* and control *GR^WT^* mice, as described above, at circadian time (CT)0 (the start of the subjective day; Supplemental Fig. 1), with either Dex or saline pretreatment. Right lung lobes were snap-frozen on dry ice for RNA extraction. After RNA purification (RNeasy Mini Kit; Qiagen, Hilden, Germany), 5 samples/group were analyzed with the nCounter Mouse Inflammation v.2 Assay (XT-CSO-MIN2-12; Nanostring Technologies, Seattle, WA, USA). Gene counts were normalized using nSolver software (Nanostring Technologies), and normalized reads were analyzed by 2-way ANOVA, *P-*value–adjusted with a false-discovery rate of 0.05. Hierarchical clustering was performed in genes showing significant drug effects with Morpheus software *(https://software.broadinstitute.org/morpheus/)*.

### RNA sequencing

RNA was extracted with the PicoPure RNA Isolation Kit (Thermo Fisher Scientific), with RNA integrity checked with the RNA 6000 Pico Kit (Agilent Technologies, Santa Clara, CA, USA); 100 ng/sample (RNA integrity number, ≥6) was used for RNA sequencing. The library was constructed with TruSeq v.3 Kit (Illumina, ‎San Diego, CA, USA) by the low-input method after poly(A) selection of mRNA. After filtering and mapping, differentially expressed genes were called using DEseq2 software (23).

### Lung digestion and macrophage sorting

Mouse lungs were harvested into ice-cold Rosewell Park Memorial Institute (RPMI) 1640 medium and minced into fine pieces using scissors before digestion in 1 ml Liberase TM (0.156 mg/ml; F. Hoffman-La Roche) with 0.01 mg/ml DNase (F. Hoffman-La Roche) for 30 min at room temperature. The reaction was stopped with an equal volume of 5 mM EDTA solution. The resulting solution was applied onto a 70-µm cell strainer, and tissue pieces were ground with a syringe head. For lung tissue macrophages, near-infrared, fixable, live/dead stain (1:1000, L10119; Thermo Fisher Scientific), anti-CD45 (as previously described), anti-Siglec-F phycoerythrin (1:200, BD Pharmingen brand clone E50-2440, 552126; BD Bioscience), and anti-CD11c (as previously described) antibodies were used. Macrophages were defined as CD45^+^Siglec-F^+^Cd11c^+^.

### Peritoneal macrophage isolation

Peritoneal macrophages were isolated, as previously described by Gibbs *et al.* (20). Cells were spun, resuspended in DMEM with 10% fetal bovine serum, and left to attach for 1–2 h on tissue culture–grade Petri dishes. Cells were then washed with PBS, and attached cells were used as macrophages.

### Western blot

GR Western blot was performed with 20 µg peritoneal macrophage protein lysate, obtained with Tissue Protein Extraction Reagent (T-PER; Thermo Fisher Scientific) and quantified by BCA assay (MilliporeSigma). Primary antibodies were GR (1:1000, clone M-20, sc-1004; Santa Cruz Biotechnology) and β-tubulin (1:10,000, clone AA2, 05-661; MilliporeSigma). Secondary antibodies were goat anti-rabbit IRDye 800CW and goat anti-mouse IRDye 680RD (926-32211 and 926-68070, 1:10,000 dilution for both; Li-Cor Biosciences, Lincoln, NE, USA). The membrane was scanned with Odyssey CLx (Li-Cor Biosciences).

### Statistics

Statistical analysis was performed with Prism software v.6 (GraphPad Software, La Jolla, CA, USA). Results are presented as means ± se, unless otherwise stated. Comparisons of 2 groups were performed with the Students’ *t* test, and 1-way ANOVA with Tukey’s correction was used to compare multiple groups as appropriate. Where multiple variables were investigated, analysis was performed *via* 2-way ANOVA with Sidak’s multiple comparisons test. In some cases (detailed in figure legends), outlier analysis was performed with the Grubbs’ test, and outliers removed before further analysis.

## RESULTS

### Rhythmic circulating Gcs are not required for time-of-day variation in pulmonary LPS responses

**Figure 1:**
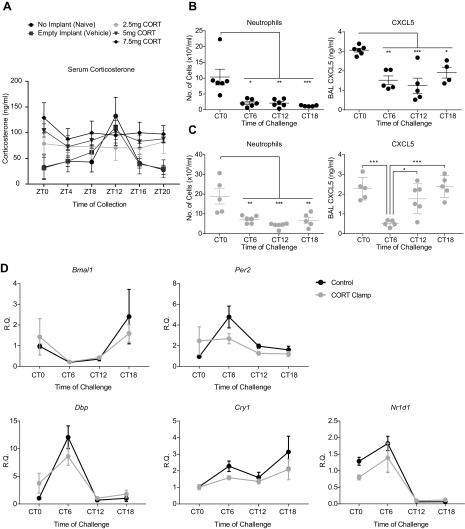
Time-of-day variation in pulmonary LPS response is retained despite corticosterone clamp. *A*) Dose–responses used to establish optimal corticosterone (CORT) clamp concentration. Corticosterone concentration in serum samples from tail blood taken at the indicated time points, *n* = 5–7. Analysis was performed by 2-way ANOVA with Sidak’s multiple comparisons test between time points, and a significant main effect of treatment was observed. Individual dose plots are shown in Supplemental Fig. 2; a dose of 2.5 mg was used in subsequent experiments. *B*, *C*) Inflammatory measurements 5 h after nebulized LPS exposure at the indicated time points. Neutrophil counts in BAL fluid and BAL CXCL5 concentration in control animals (*B*) and CORT-clamped animals (*C*), median age 12 wk, *n* = 4–6/group. Analysis using Grubbs’ test revealed 3 outliers in the CXCL5 results in *C* (1 each from CT0, CT6, and CT18), which were removed before further analysis. Analysis was performed by 1-way ANOVA and *post hoc* tests with Tukey’s correction. Asterisk denotes significance from CT0 time point, except where indicated. *D*) mRNA expression of a panel of clock genes in whole lung of CORT-clamped and control animals after LPS challenge. Analysis was performed *via* 2-way ANOVA; all genes showed a significant effect of time but no differences between treatments. Data represent means ± se. *P < 0.05, **P < 0.01, ***P < 0.001, ****P < 0.0001.

In mice, adrenalectomy only partially ablates the endogenous rhythm of corticosteroid because a residual, low-amplitude oscillation persists (3). To test the importance of circulating corticosterone as a timing signal, C57BL/6J mice were implanted with slow-release corticosterone (“clamp”), which, *via* feedback repression, eliminates the rhythmic secretion of endogenous Gc. Serum corticosterone concentration was measured 1 wk afterward at 6 different time points for animals housed in light/dark cycles (ZT), where ZT0 is lights on, and ZT12 is lights off; sampling times were ZT0, 4, 8 12, 16, and 20). We employed a range of doses, from which we selected a dose (2.5 mg implant) that elicited a flat, unchanging, Gc profile and was midpoint between the peak and trough of the endogenous signal of unimplanted animals (**Fig. 1*A*** and see Supplemental Fig. 2*A* for individual plots by dose).

Based on this “midpoint” dose, we treated clamped and control mice with nebulized LPS at 4 time points (CT0, start of subjective day; CT6, middle of subjective day; CT12, start of subjective night; and CT18, middle of subjective night), with BAL fluid collected 5 h later to analyze leukocyte infiltration and CXCL5 concentration. Corticosterone concentrations showed significant time-of-day variation in control mice, but no temporal variation was observed in clamped animals (Supplemental Fig. 2*B*). We observed strong rhythmic neutrophil infiltration in control mice, as previously reported by Gibbs *et al.* (3), and also in clamped mice, with similar amplitude and phasing. We have previously described circadian amplitude changes of a key pulmonary chemokine CXCL5 involved in rhythmic inflammatory responses (3); this showed robust circadian changes in both groups of animals (Fig. 1*B*, *C*). Because Gcs are known to act as potent resetting agents for the circadian clock, we tested whether the clamp procedure altered expression of underlying circadian clock gene expression in whole lung. There was a significant effect of time on expression, but the phase and amplitude of expression was unaltered by the treatment (Fig. 1*D*).

### GR deletion in airway epithelium disrupts Dex-induced synchronization in the bronchioles but not the parenchyma

**Figure 2:**
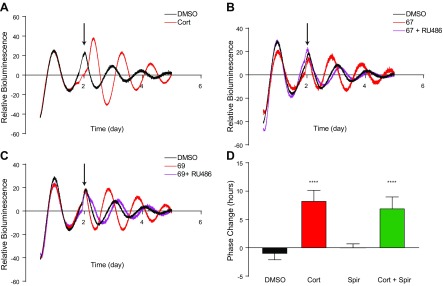
Endogenous Gcs act through GR to modulate lung rhythmicity. *A*) Lung slices were taken from PER2::LUC mice and bioluminescence was recorded before and after treatment with Cort (100 nM; arrow denotes treatment time). DMSO was used as a control in all experiments; data are representative of 3–7 experiments. *B*, *C*) Sections were treated with GSK67a (10 nM) and RU486 (1 µM; *B*) or GSK69 (10 nM) and RU486 (*C*); data are representative of 3–7 experiments. *D*) Sections treated with Cort (100 nM), the MR-specific antagonist, spironolactone (Spir; 1 µM), or both Cort and Spir. Phase change was calculated relative to an untreated peak (positive being a delay, and negative being an advance). Analysis was performed with 1-way ANOVA and *post hoc* tests with Tukey’s correction, *n* = 3–7. Data represent means ± se. ****P < 0.0001 compared with DMSO control.

As loss of the circadian rhythm in serum corticosterone had no effect on circadian control of pulmonary inflammation, we next sought to examine the role of its receptors. Corticosteroids bind both the MR and GR with equal affinity. We, thus, studied which receptor had a dominant effect in mediating Gc signaling to the pulmonary circadian oscillator. For this, we employed ectopic lung slices derived from PER2::LUC reporter mice (18) and monitored them using whole-field bioluminescence (19). We treated explants with corticosterone at the peak of the circadian cycle of PER2::LUC bioluminescence. Corticosterone administration resulted in increased amplitude of the oscillations, along with a phase change (**Fig. 2*A***). We next employed novel GR-specific, nonsteroidal ligands 67 and 69 (21), which are known not to have MR action and are highly selective GR ligands. This revealed marked PER2::LUC resetting responses, which were blocked by the GR antagonist RU486 (Fig. 2*B*, *C*). In contrast, the MR antagonist spironolactone did not phase shift the PER2::LUC oscillation and did not block the action of Gcs (Fig. 2*D*). Collectively, these data clearly demonstrate that GR is the primary receptor involved in synchronization of the circadian clock by endogenous Gcs in the lung.

**Figure 3:**
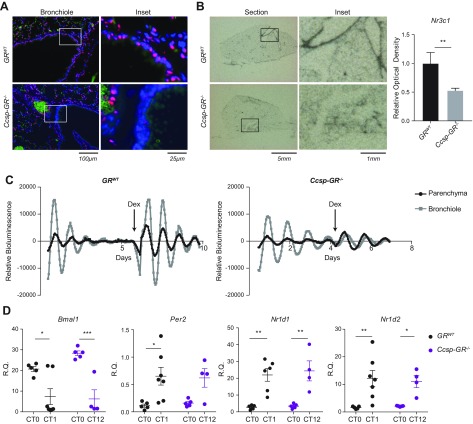
Deletion of GR in airway epithelial cells of *Ccsp-GR^−/−^* mice is associated with reduced responsiveness to Dex-induced circadian synchronization. *A*) Immunofluorescence images of lung sections from *GR^WT^* (top) and *Ccsp-GR^−/−^* mice (bottom) stained for GR (red) and cell nuclei (DAPI). Green indicates background/auto fluorescence. *B*) Images of lung sections from *GR^WT^* (top) and *Ccsp-GR^−/−^* mice (bottom) probed for GR mRNA *via* *in situ* hybridization with relative quantification of GR (*Nr3c1*) mRNA abundance from multiple lung sections (*n* = 4/genotype, analyzed with 2-tailed Student’s *t* test). *C*) PER2-LUC bioluminescence (photon counts/min) over multiple days from *GR^WT^* (left) and *Ccsp-GR^−/−^* (right) lung sections. Dex was administered at the indicated time (arrow). Plot is representative of 3 independent replicates. *D*) Relative quantification of *Bmal1*, *Per2*, *Nr1d1*, and *Nr1d2* gene expression in whole lung (*n* = 4–7, 2-way ANOVA with Sidak’s multiple comparisons test within genotypes). For *Nr1d1*, 1 outlier was removed from the *GR^WT^* group at CT12 after using the Grubbs’ test to detect outliers. Data represent means ± se. *P < 0.05, **P < 0.01, ***P < 0.001.

Having established that, we next addressed the role of the GR in pulmonary airway epithelial cells in mediating inflammatory responses, and generated a transgenic mouse line that lacked GR in the airway epithelium (*Ccsp-GR^−/−^*). Mice were born with expected Mendelian ratios and survived into adulthood without apparent health issues, and no apparent pulmonary morphologic changes were observed. Using immunohistochemistry, we stained for pulmonary GR expression, which revealed a loss of expression specific to the bronchial epithelium, but no obvious alteration in expression in adjacent parenchymal structures (**Fig. 3*A***). Additionally, we checked expression of the transcript in tissue sections using *in situ* hybridization, which also revealed a clear loss of expression in conducting airways (Fig. 3*B*). Thus, the *Ccsp-GR^−/−^* construct effectively targets those cells in the lung. Histologic analysis of lung sections revealed no obvious morphologic or developmental differences.

To examine the effects of GR loss upon circadian rhythmicity of the lung, the strain was then bred onto the *mPer2^Luc^* background (18). Lung sections from these mice were harvested and tested for responsiveness to synchronization by the GR agonist Dex. We recorded luciferase activity with a bioluminescence camera to compare signals from bronchiolar and parenchymal regions (Fig. 3*C* and Supplemental Videos 1 and 2). Tissues were exposed to recording medium (time 0) and, when dampened to a nonrhythmic state, treated with Dex. This had a powerful resetting effect on PER2::LUC expression in both bronchial and parenchymal regions in *GR^WT^* mice. In contrast, Dex did not reset bronchiolar tissues derived from *Ccsp-GR^−/−^* animals, but we did observe robust resetting responses in adjacent parenchymal tissues (Fig. 3*C*). Thus, the effect of GR targeting is specific to cell type with in the ectopic lung slice. Analysis of whole-lung tissue from *GR^WT^* and *Ccsp-GR^−/−^* mice showed no significant effect of loss of GR on overall expression of *Bmal1*, *Per2*, and *Nr1d1/2* (Fig. 3*D*). We next assessed the potential effect of such targeting on the operation of the circadian gene complex within GR-targeted cells. As gene expression in whole lung may mask critical changes in airway epithelial cells, we employed laser-capture microscopy to harvest airway epithelial cells. These were collected from mice free-running in darkness every 4 h for 44 h to produce a 2-cycle time series in airway epithelial cells. This revealed robust oscillations in multiple components of the circadian clock. Although an effect of genotype was observed upon *Dbp* and *Nr1d1*, with *Ccsp-GR^−/−^* mice showing elevated expression at the peaks of the cycle, that did not affect expression of their target genes *Per2*, *Cry1*, and *Bmal1*, respectively (Supplemental Fig. 3), suggesting these minor amplitude differences lack significant functional effect on the core clockwork. Thus, Gc-mediated resetting of the circadian clockwork depends critically on GR, and not MR, in target cells, but loss of GR does not affect free-running endogenous oscillations of the circadian clock gene complex in the targeted cells.

### Deletion of GR in airway epithelium disrupts CXCL5 rhythmicity but not rhythmic neutrophilia

**Figure 4:**
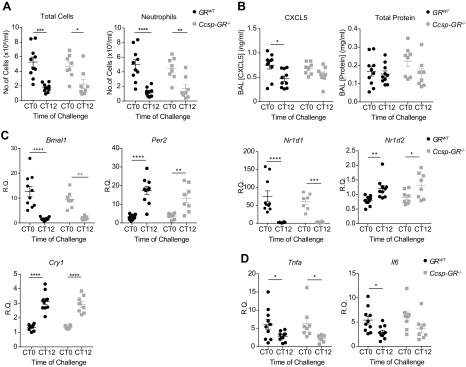
Loss of GR in the bronchiolar epithelium does not affect circadian gating of neutrophilia after LPS challenge. *GR^WT^* and *Ccsp-GR^−/−^* mice (median age, 10 wk) exposed to nebulized LPS at CT0 or CT12, with samples collected 5 h later. *A*) Quantification of total cells (left) and neutrophils (right) in BAL fluid. *B*) BAL CXCL5 (left) and total protein (right) concentration. *C*, *D*) Expression of clock genes (*C*) and inflammatory genes (*D*) in lung homogenate after challenge. For all panels, *n* = 8–10; data were analyzed with 2-way ANOVA, followed by Sidak’s multiple comparisons test for effects within genotype. Data represent means ± se.*P < 0.05, **P < 0.01, ***P < 0.001, ****P < 0.0001.

We have previously identified rhythmic GR binding to the *Cxcl5* promoter in lung as a key component in conferring time-of-day changes in inflammatory responses, and this was abolished when the circadian clock element Bmal1 was disrupted in pulmonary epithelial cells (3). To test the role of GR, we exposed *Ccsp-GR^−/−^* mice to nebulized LPS at CT0 and CT12 and assessed temporal variation in total leukocyte numbers and neutrophil infiltration. This revealed marked time-of-day effects, as previously reported in Gibbs *et al.* (3), but no effect of genotype (**Fig. 4*A***). In contrast, chemokine CXCL5 responses to LPS exhibited marked time-of-day differences in *GR^WT^* mice, also compatible with the previous report (3), but this time-of-day gating was lost in the *Ccsp-GR^−/−^* mice (Fig. 4*B*). Further, we observed no differences in total protein concentration of BAL, a biomarker for epithelial permeability. In whole lung, expression patterns of the clock genes *Bmal1*, *Per2*, *Cry1*, *Nr1d1*, and *Nr1d2* (Fig. 4*C*) and inflammatory marker genes *Tnfa* and *Il6* (Fig. 4*D*) in response to LPS were unaltered in *Ccsp-GR^−/−^* mice.

### Deletion of GR in airway epithelium does not affect anti-inflammatory effects of Dex

**Figure 5:**
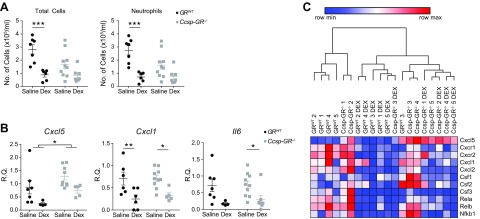
*Ccsp-GR^−/−^* mice retain sensitivity to anti-inflammatory effects of Dex during LPS challenge. *GR^WT^* and *Ccsp-GR^−/−^* mice (median age, 12 wk) were exposed to nebulized LPS at CT0 and culled 5 h later. The intraperitoneal injection of either Dex (1 mg/kg) or saline (vehicle) took place 1 h before LPS exposure. *A*, *B*) Quantification (*A*) of total cells (left) and neutrophils (right) in BAL fluid. Expression (*B*) of *Cxcl5*, *Cxcl1*, and *Il6* in lung homogenate after challenge. *n* = 6–10, and data were analyzed with 2-way ANOVA, followed by Sidak’s multiple comparisons test for effects within genotype. *C*) Hierarchical clustering of proinflammatory cytokines/chemokines from Nanostring analysis (see Supplemental Fig. 6 for full heat map of Dex-responsive genes, and Supplemental Table 1 for list of all genes analyzed). Data represent means ± se. *P < 0.05, **P < 0.01, ***P < 0.001.

Gcs are known to reduce neutrophil influx in response to nebulized LPS (3). To test whether GR in the epithelial cells is required, *Ccsp-GR^−/−^* and *GR^WT^* control mice were compared at CT0 (the time of maximal neutrophil influx), with or without pretreatment with Dex (1 mg/kg, i.p.). Dex treatment significantly reduced total cell count and neutrophil number in BAL for both *GR^WT^* and *Ccsp-GR^−/−^* mice with no significant interactions between genotype and treatment (**Fig. 5*A***), but a *post hoc* analysis indicated effects in *GR^WT^* animals. Analysis of gene expression in lung homogenate showed a significant reduction in *Cxcl5* expression after Dex in both groups but with markedly elevated baseline expression in *Ccsp-GR^−/−^* mice. Additionally, Dex also suppressed known Gc target genes *Cxcl1* and *Il6* in an equivalent manner (Fig. 5*B*) but had no effect upon a panel of clock genes (Supplemental Fig. 4). Equivalent experiments performed at CT12 (the time of minimal neutrophil influx) showed no significant effects of Dex or genotype upon cell influx or CXCL5 concentration (Supplemental Fig. 5).

To extend assessment of potential GR targets, we used an inflammatory gene expression array panel (nCounter Mouse Inflammation V2, XT-CSO-MIN2-12; Nanostring Technologies) covering 248 genes and tested Dex responses in LPS-treated mice. Both trans-repression and trans-activation GR targets were represented in the array (*e.g.*, *Csf3*, *Il23a*, *Il1b*, and *Retnla*, *Chil3l3*, *Tgfb3*, respectively). Approximately one half of the panel (114 genes) showed significant responses to Dex (Supplemental Table 1). An unbiased, hierarchical clustering protocol revealed major effects of Dex but little effect of genotype (Supplemental Fig. 6). Strikingly, *Cxcl5* was the marked exception and the only transcript with altered responses in *Ccsp-GR^−/−^* lung tissue (Fig. 5*C* and Supplemental Table 1).

### Time-of-day difference in pulmonary *Cxcl5* and *Cxcl15* expression is ablated in *Ccsp-GR^−/−^* mice in steady-state conditions

**Figure 6:**
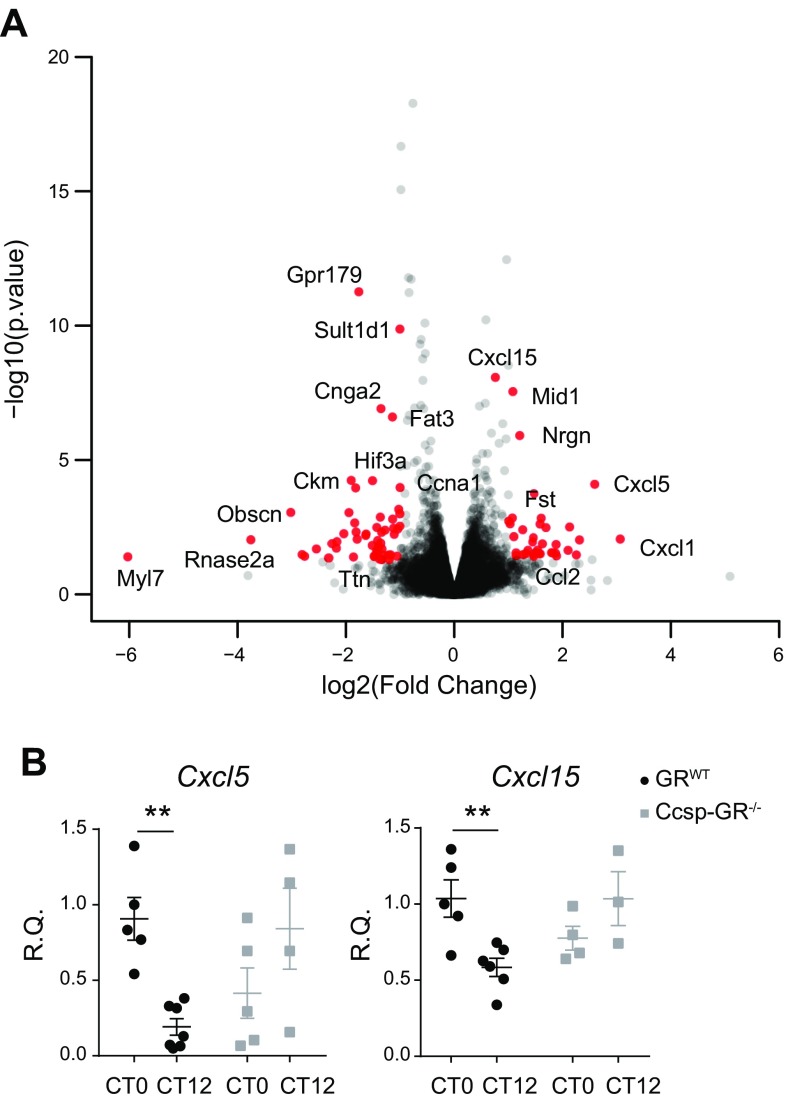
Airway epithelium-specific GR loss affects gene expression during homeostasis. RNA-sequencing from laser-microdissected distal bronchiolar epithelial cells at ZT14. *A*) Volcano plot of gene expression in bronchiolar epithelial cells from *Ccsp-GR^−/−^* mice relative to *GR^WT^* littermate controls. *B*) Assessment of rhythmic expression of *Cxcl5* and *Cxcl15* in whole lung harvested at the indicated time points (*n* = 3–6, 2-way ANOVA with Sidak’s multiple comparisons test within genotype). Data represent means ± se. **P < 0.01.

Deletion of bronchial epithelial GR did not significantly alter neutrophilia in lung. To assess wider potential effect, we undertook RNA sequencing analysis of bronchial epithelium, collected by laser microdissection of lung samples frozen at ZT14, timed for 2 h after the endogenous corticosterone peak; 111 differentially expressed genes were found with a criteria of *P* < 0.01 and a fold change ≥1.5 or ≤1.5, including *Cxcl5*, *Cxcl1*, and *Cxcl15*, which were all increased in *Ccsp-GR^−/−^* samples (**Fig. 6*A***).

Gene-ontology analysis of the RNA sequencing data set revealed significant changes in inflammatory response terms, as expected, along with lipid metabolic processes and response to hypoxia (**Table 1**). However, those analyses were performed under light/dark conditions, so we tested whether similar effects were seen under true circadian conditions of continuous darkness. Here, we also saw a loss of circadian gating responses of *Cxcl5* and *Cxcl15* in *Ccsp-GR^−/−^* mice (Fig. 6*B*).

**TABLE 1:** Analysis of biologic pathways affected by epithelium-specific GR loss

GO term	*P*
Response to hypoxia	1.5E−4
Lipid metabolic process	6.2E−4
Negative regulation of inflammatory process	5.7E−3
Positive regulation of transcription from RNA PolII promoter	7.8E−3
Cellular response to TNF	1.1E−2
Cellular response to cyclohexamide	1.2E−2
Inflammatory response	1.4E−2

Gene Ontology (GO; *http://www.geneontology.org/*)term biologic pathway–enrichment analysis of differentially expressed genes in distal bronchiolar epithelial cells harvested at ZT14 from *Ccsp-GR^−/−^* mice and *GR^WT^* littermate controls (see Fig. 6*A* for volcano plot of gene expression).

### Myeloid GR deletion does not alter circadian variation in pulmonary LPS response or Dex sensitivity

We next tested the role of myeloid cells as critical responders in LPS-induced inflammation. To target macrophage populations, we generated *LysM-GR^−/−^* mice. Successful deletion of GR was confirmed in protein from peritoneal macrophages (Supplemental Fig. 7*A*) and also in RNA from pulmonary macrophages, confirming that GR was deleted in both BAL and tissue-resident macrophage populations (Supplemental Fig. 7*B*). Although cultured *LysM-GR^−/−^* peritoneal macrophages were circadian rhythmic, they were no longer sensitive to Dex-induced resynchronization (Supplemental Fig. 7*C*).

**Figure 7:**
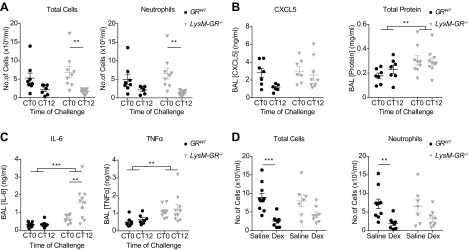
*LysM-GR^−/−^* mice retain time of day variation in response to nebulized LPS and Dex sensitivity. *GR^WT^* and *LysM-GR^−/−^* mice (median age, 12 wk) were exposed to nebulized LPS at the indicated time points and culled 5 h later. *A*) Quantification of total cells (left) and neutrophils (right) in BAL fluid. *B*) Concentrations of CXCL5 (left) and total protein (right) in BAL. *C*) Concentrations of IL-6 (left) and TNF-α (right) in BAL. *D*) The intraperitoneal injection of Dex (1 mg/kg) or saline (vehicle) took place 1 h before LPS exposure at CT0. BAL was collected 5 h after LPS challenge, and total cells (left) and neutrophils (right) were quantified. For all panels, *n* = 7–10; data were analyzed with 2-way ANOVA and Sidak’s multiple comparisons test within genotypes. Data represent means ± se. **P < 0.01, ***P < 0.001.

To examine the effect of myeloid cell GR disruption on time-of-day variation in pulmonary LPS responses, *LysM-GR^−/−^* mice were exposed to nebulized LPS at CT0 and CT12. Time-of-day differences were observed for total leukocyte counts and BAL neutrophils in both *LysM-GR^−/−^* and *GR^WT^* littermate control mice (**Fig. 7*A***). In contrast, there was no time-of-day variation of BAL CXCL5, and total protein concentration in BAL was significantly elevated in *LysM-GR^−/−^* mice (Fig. 7*B*), indicative of disrupted epithelial barrier function. In addition, inflammatory cytokines IL-6 and TNF-α were significantly increased (Fig. 7*C*).

Finally, to test whether myeloid cell GR mediated Dex suppression of the pulmonary LPS responses, *LysM-GR^−/−^* and control mice were treated with nebulized LPS at CT0 with or without Dex pretreatment (1 mg/kg, i.p.). Analysis of the cellular content of BAL fluid showed retention of Dex sensitivity in *LysM-GR^−/−^* animals, with comparably fewer BAL cells after Dex treatment in both genotypes (Fig. 7*D*).

## DISCUSSION

Gcs are very widely used to treat inflammatory diseases, and natural, endogenous Gcs are the principal anti-inflammatory response system *in vivo* (24). Despite such widespread application, it remains unclear which are the main target cells and how the response to Gc varies. Endogenous Gc production is pulsatile, with a superimposed circadian rhythm (25), but this aspect of physiology is not considered in therapeutic development or application. The cross-coupling of Gc action to the circadian clock is extensive, with Gc serving as regulators of circadian clock phase. *In vitro* application of Gc to cells in culture potently resets their clock (6). Less is known of how Gc action and the circadian clock interact *in vivo*, but adrenalectomy reduces the response time for animals to complete a phase shift to a new lighting schedule (26, 27), and circadian liver function has been linked to Gc action (7). We have previously noted that therapeutic Gc use in animal models of lung inflammation was differentially effective by time of day, and at a molecular level, we identified a circuit linking the circadian clock *via* *Bmal1* and *Rev-erbα* to regulation of neutrophilic inflammation through the airway epithelial GR (3, 28). However, in those studies, we did not distinguish between a circadian signal from the adrenal production of corticosterone, and circadian control of the ligand-activated GR.

**Figure 8:**
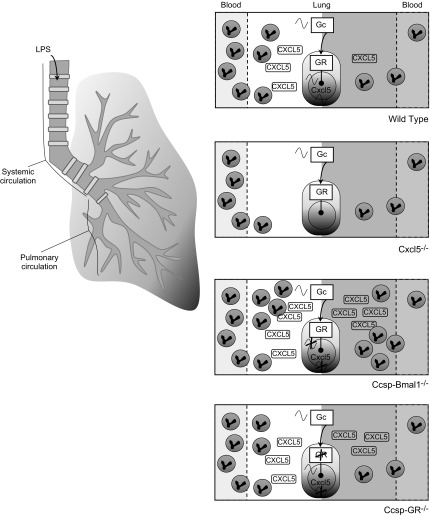
The gated LPS response. WT: Upon stimulation with LPS, inflammatory signaling pathways in the lung are engaged, resulting in production of proinflammatory chemokines and neutrophil influx through the pulmonary circulation (42, 43). In WT animals, the clock regulates access to modulatory regions of chromatin, including an enhancer region in the *Cxcl5* sequence to which GR has been shown to bind with inhibitory effects upon transcription. At CT12, GR binding is permitted, and cell influx is suppressed relative to the challenge at CT0. A strong signal (CXCL5) combined with more neutrophils in the circulation during the day (34) leads to an oscillation in infiltrating cell count (3). *Cxcl5^−/−^*: CXCL5 predominates in driving neutrophil influx to the alveolar space in the nebulized LPS model (30). Mice can no longer produce *Cxcl5* upon LPS stimulation, resulting in low numbers of invading neutrophils at both time points. Some cells are still found in lavage fluid, indicating successful migration without a CXCL5 signal (3). *Ccsp-Bmal1^−/−^*: Without *Bmal1* in the bronchial epithelial cells, the inhibitory binding of GR no longer occurs at CT12 and gating of CXCL5 production is lost. Production of CXCL5 is enhanced and increased neutrophilia occurs at both time points without a day/night variation in infiltrating cell number (3). *Ccsp-GR^−/−^*: Without GR in the bronchial epithelial cells, the inhibitory binding of GR no longer occurs at CT12, and gating of CXCL5 production is lost despite the presence of an intact circadian clock. However, an intact oscillation in cell influx remains. A nonrhythmic signal (CXCL5), combined with oscillations in neutrophil count in the bloodstream (34), still results in an oscillation in infiltrating cell count.

Therefore, our initial studies dissociated the timing signal in serum corticosterone, by using a clamp. That did not alter the pattern of CXCL5 production or BAL immune cell infiltration. GR itself may be directly regulated by the circadian clock and independent of a rhythmic ligand signal, perhaps through modification by CLOCK acetyltransferase activity (23) or protein interaction with the cryptochrome transcription factors (8). Accordingly, we moved to investigate the GR itself. Because corticosterone can activate both the GR and the closely related MR, we needed to establish which receptor to target. We used lung slice cultures and were able to see a marked clock phase shift with corticosterone and also with GSK67 and 69, 2 nonsteroidal GR agonists with very high potency and specificity (21). Importantly, RU486 was able to antagonize the GSK67/69 effects, but the MR antagonist had no effect on corticosterone-induced resetting. Therefore, we targeted deletion of the GR to the airway epithelium (*Ccsp-GR^−/−^*). Despite the crucial role of GR in lung development and the effects of GR loss in other lung cell populations (29), mice were born in the expected Mendelian ratio and lacked apparent lung pathology. We saw no change in the circadian rhythm of LPS-induced neutrophilic inflammation, but strikingly, we did abolish the rhythm in CXCL5. That discord between neutrophil chemokine and neutrophil influx was surprising, given that CXCL5 is the dominant regulator of neutrophil influx to the alveolar space in this model (30) and is strikingly different to the phenotype seen in *Ccsp-Bmal1^−/−^* animals, where both CXCL5 and neutrophil influx lost oscillation with greatly increased production of CXCL5 along with elevated pulmonary neutrophilia (3) (**Fig. 8**). This reveals that epithelial GR is required to transmit the time of day signal to *Cxcl5* expression but that additional pathways and factors are operating to gate neutrophil infiltration to the lung.

It is becoming increasingly apparent that leukocyte trafficking to tissues is under rhythmic regulation at multiple levels (31, 32), and neutrophil mobility is no exception. Neutrophil content in the bloodstream peaks at ZT5, potentially providing a larger pool of circulating cells for chemokine signals to reach if inflammatory stimuli are administered at the start of the day (reviewed in Scheiermann *et al*. 33). However, neutrophils also age in the circulation and up-regulate CXCR4, which drives rhythmic homing to the bone marrow (34). In addition, neutrophil function is not necessarily in concordance with neutrophil numbers. We have previously reported that the up-regulation of neutrophil influx in mice lacking a clock in the pulmonary epithelium (*Ccsp-Bmal1^−/−^*) does not confer an advantage in bacterial clearance after *Streptococcus pneumoniae* infection (3). Here, we also report no change in total BAL protein concentrations with neutrophil number, indicating that rhythmic changes in neutrophilia are not associated with rhythmic changes in general lung permeability in this model. That is interesting because lung injury after LPS inhalation was previously shown to be neutrophil dependent (35). Neutrophils may, therefore, also exhibit temporal changes in their sensitivity to chemokine signals and their effector functions, independent of their number in circulation or in the alveolar space.

Because our *Ccsp-GR^−/−^* transgenic mice serve as an excellent tool for studying the role of GR in the airway epithelium, we tested a therapeutic application of Dex. Surprisingly, Dex is still anti-inflammatory with many classic inflammatory genes, such as *Il6* and *Cxcl1*, suppressed in both WT and *Ccsp-GR^−/−^* mice. In a broader panel of 248 genes, *Cxcl5* showed a genotype effect, consistent with the pulmonary epithelium being a major source of this Gc-repressed chemokine. However, additional neutrophil chemoattractants, such as *Cxcl1* (KC) and *Cxcl2* (MIP-2), along with the receptors *Cxcr1* and *Cxcr2* were unaffected by loss of epithelial GR, and these were comparably affected by Dex treatment. The colony-stimulating factors G-CSF (*Csf3*) and GM-CSF (*Csf2*), which regulate chemokine production and neutrophil survival and accumulation in the airways (36–38), were also unaffected by genotype. These factors are produced by multiple cell types within the lung and show that our targeting of the bronchial epithelial cells results in specific changes in gene expression in the nebulized LPS model without significant effects upon chemokine production in the broader environment. It has recently been shown that GR agonism can have differential effects in the lung depending upon cell type and location and that endogenous Gcs, acting as partial agonists, can inhibit the effects of synthetic full agonists, such as Dex (39). Therefore, the selective ablation of the GR in a subset of epithelial cells may not have a significant effect upon a complex and broad response, such as innate inflammation but, instead, reveals the very specific nature of GR regulation of *Cxcl5* in the bronchial epithelial cell and highlights the dominant role of the clock in these cells driving rhythmic physiology and pathology in the lung (3, 19, 28).

Because airway macrophages are the other major cell sensing and responding to pathogens entering the lung, we also created mice that lacked the macrophage GR (*LysM-GR^−/−^*). Again, we were surprised to see a very minor phenotype reaction, either in response to inflammatory challenge or anti-inflammatory Dex treatment. The retention of Dex sensitivity in our studies differs from the reports of Bhattacharyya *et al.* (40) and of Vettorazzi *et al.* (41), who found an impaired anti-inflammatory response to Dex in *LysM-GR^−/−^* mice. GR inhibition of macrophage p38 stress-activated protein kinase signaling was shown to be crucial for survival in LPS-induced sepsis (40). In another model, a circuit was identified that required macrophage Gc signaling to prime local induction of sphingosine kinase 1 (SphK1) in adjacent endothelial cells (41). The major difference between these 2 studies, which showed a requirement for macrophage GR in regulating the inflammatory reaction, and our study, which suggests that macrophage GR action was dispensable, was the mode of LPS administration and the severity of the challenge. In our nebulized LPS model, there is minimal systemic response, and, therefore, the absence of GR within the circulating myeloid cell population was not tested, in contrast to the 2 earlier studies, in which a significant systemic effect was a major feature of the model. Our data suggest that, in the context of a localized pulmonary inflammation, Gcs can mediate anti-inflammatory effects acting through multiple cell types, with sufficient redundancy to compensate for loss in 1 cell type alone.

In summary, although bronchial epithelial GR is required for rhythmic pulmonary *Cxcl5* expression, loss of bronchial epithelial GR does not impair the rhythmic variation in pulmonary neutrophilic inflammation that we have previously characterized. Therefore, we were able to dissociate the clock control of CXCL5 from clock control of neutrophilic infiltration. In our model of acute, localized pulmonary inflammation, the epithelial GR was not required for the anti-inflammatory action of therapeutically administered Dex, suggesting additional cell types mediate that important effect. Further analysis of the myeloid lineage GR also revealed a surprisingly mild phenotype with minimal effects on lung inflammation and its therapeutic inhibition by Dex. Taken together, our studies reveal that neither airway epithelial nor macrophage GR is required for Dex-mediated suppression of nebulized LPS-induced acute lung injury.

## Supplementary Material

This article includes supplemental data. Please visit *http://www.fasebj.org* to obtain this information.

## Abbreviations

BAL: bronchoalveolar lavage
BCA: bicinchoninic acid
CCSP: club cell secretory protein
CT: circadian time
CXCL5: CXC chemokine ligand 5
Dex: dexamethasone
Gc: glucocorticoid
GR: glucocorticoid receptor
MR: mineralocorticoid receptor
qPCR: quantitative PCR
WT: wild type
ZT: zeitgeber time
