# Supplementary material for: Circadian variation in pulmonary inflammatory responses is independent of rhythmic glucocorticoid signaling in airway epithelial cells
Source: FASEB J. 2018 Jul 2;33(1):126–39. doi: 10.1096/fj.201800026RR (PMC6355062; doi:10.1096/fj.201800026RR)
Supplement: Supplementary file 1 [file fj.201800026RR.sf1.pdf]

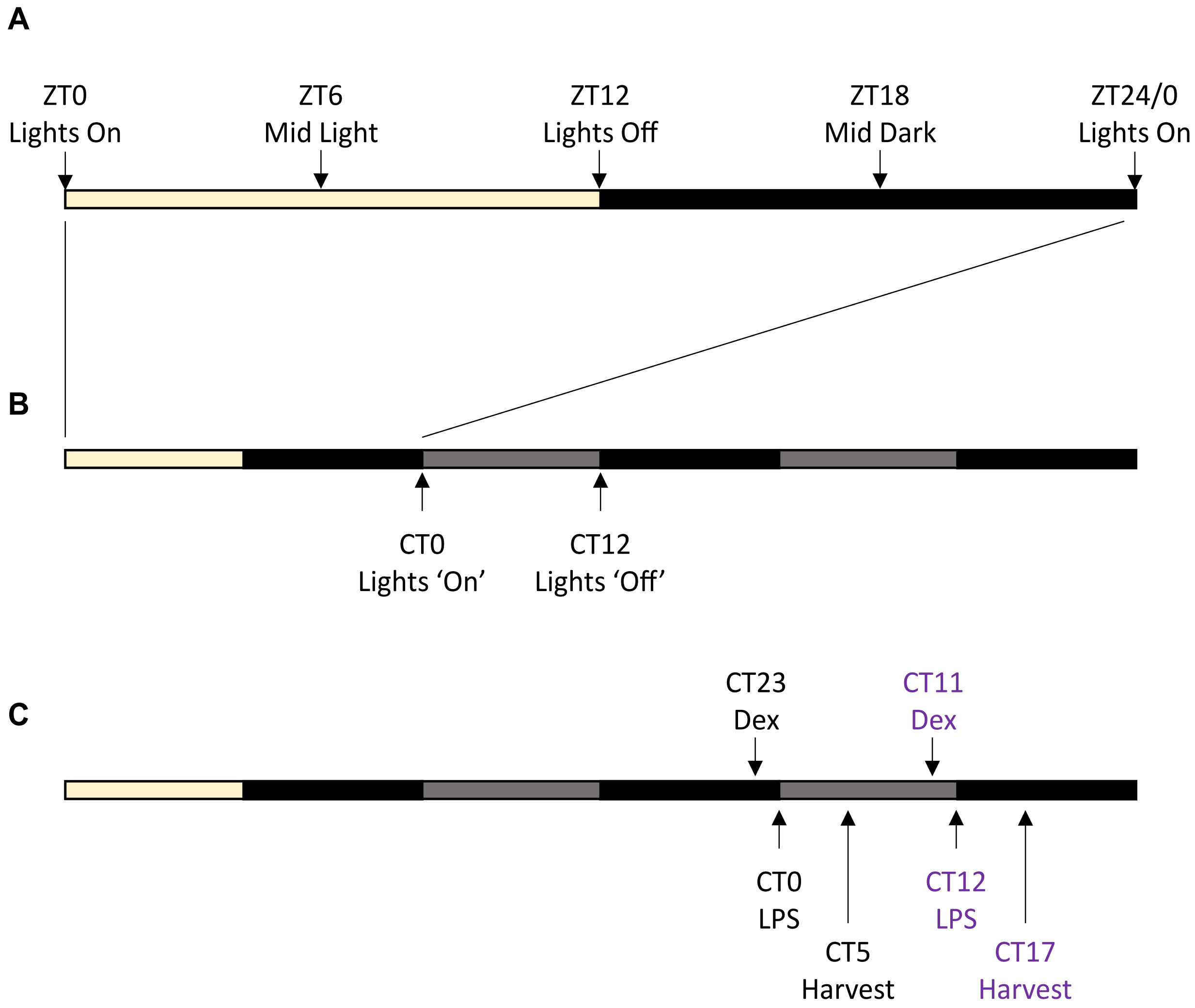

Supplemental Figure 1: Experimental protocols.

Mice were group housed with food and water ad libitum. (A) Normal lighting conditions were cycles of 12hr light: 12hr dark. In these cases, the term 'zeitgeber time' (ZT) refers to time after light onset. (B) For experiments addressing regulation by the endogenous pacemaker, mice were first transferred into constant darkness for 24hrs and then exposed to stimuli at the indicated time points (Aschoff type 2 protocol). In this situation, 'circadian time' (CT) is used, indicating the time relative to when the lights would have come on. CT0 is thus approximately equal to ZT0, except that no light signal has been given. (C) An example timing of experimental treatments and collections within this protocol. *CT – circadian time; ZT – zeitgeber time.*
