# Supplementary material for: Circadian variation in pulmonary inflammatory responses is independent of rhythmic glucocorticoid signaling in airway epithelial cells
Source: FASEB J. 2018 Jul 2;33(1):126–39. doi: 10.1096/fj.201800026RR (PMC6355062; doi:10.1096/fj.201800026RR)
Supplement: Supplementary file 2 [file fj.201800026RR.sf2.pdf]

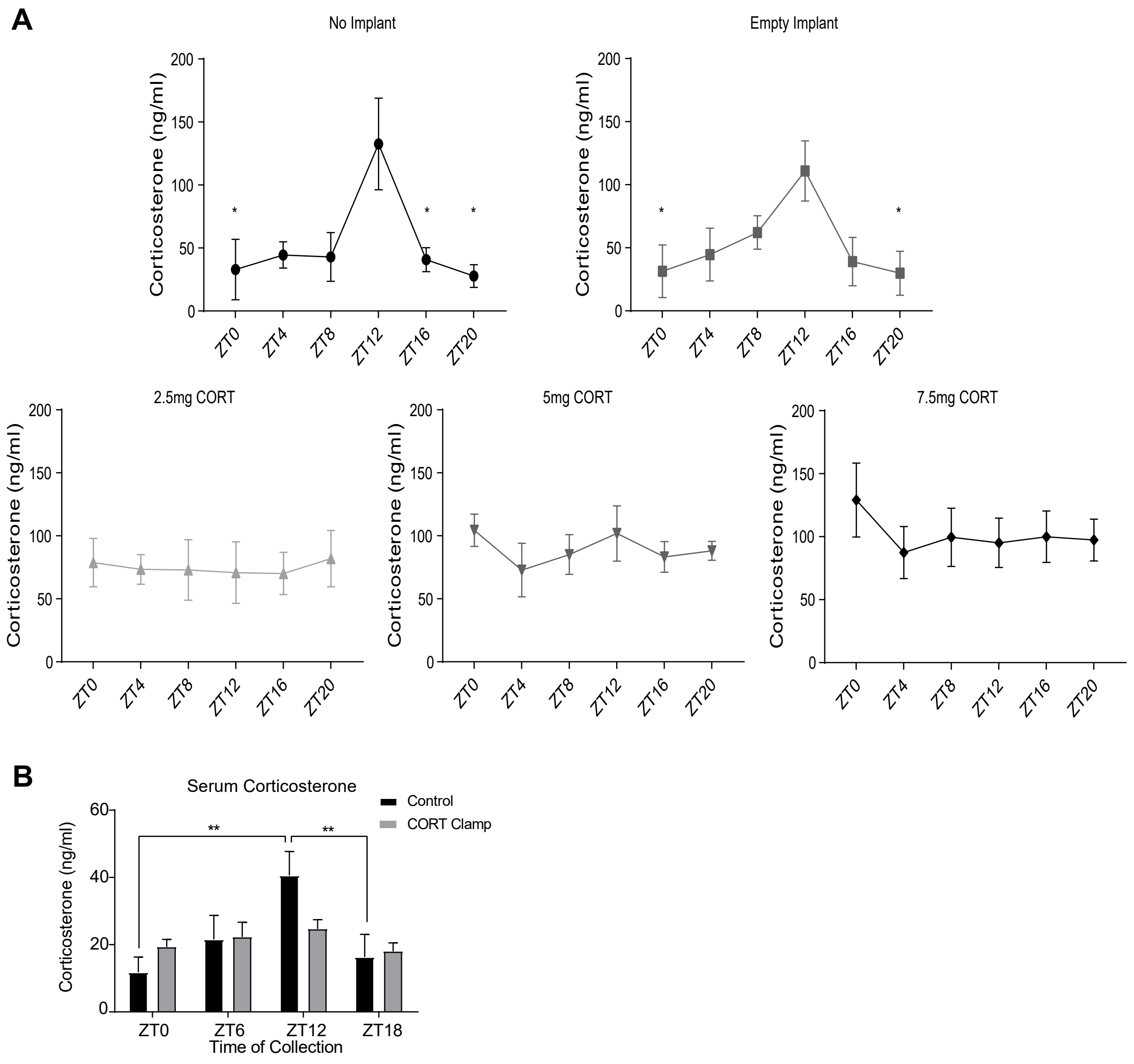

Supplemental Figure 2: Establishment of corticosterone dose for subcutaneous implant. (A) Mice were implanted with one subcutaneous sustained-release corticosterone pellet; either vehicle (n=7), 2.5mg, 5mg or 7.5mg (n=6). Naïve animals were used as an additional control (n=5). Corticosterone concentration in serum samples from tail blood taken at indicated time points. Data is taken from Figure 1A, analysed using a two-way ANOVA with Sidak's multiple comparisons test between time points (effects within-treatment). A significant effect of time was observed for both naïve and vehicle-treated animals, * denotes significant difference from CT12 values. (B) Corticosterone concentration in serum samples from tail blood taken at indicated time points, n=4-6. Analysis was performed using two-way ANOVA with Sidak's multiple comparisons test between time points (effects within-treatment). *ZT – zeitgeber time.*
