# Supplementary material for: Circadian variation in pulmonary inflammatory responses is independent of rhythmic glucocorticoid signaling in airway epithelial cells
Source: FASEB J. 2018 Jul 2;33(1):126–39. doi: 10.1096/fj.201800026RR (PMC6355062; doi:10.1096/fj.201800026RR)
Supplement: Supplementary file 3 [file fj.201800026RR.sf3.pdf]

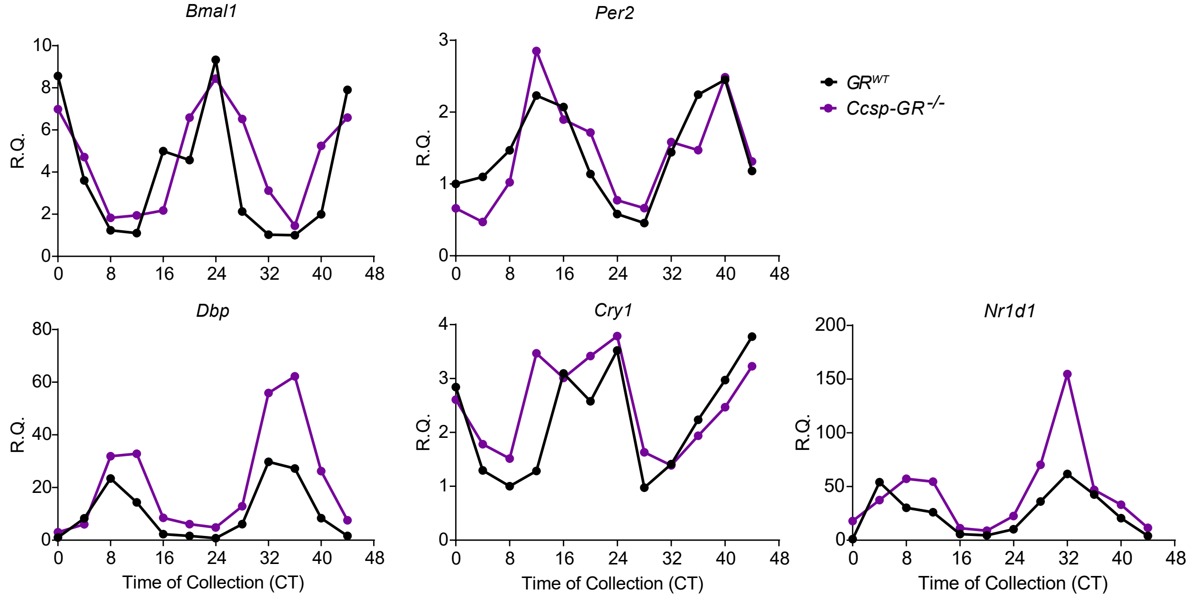

Supplemental Figure 3: Clock gene cycling in airway epithelial cells of *Ccsp-GR$^{-/-}$* and *GR^{WT}* mice.

Relative quantification of *Bmal1*, *Per2*, *Dbp*, *Cry1* and *Nr1d1* gene expression in laser-microdissected bronchial epithelium at the indicated time points (n=1/genotype/time, median age 12 weeks). Two-way ANOVA without replication was used to test main effects and showed a significant effect of time for all genes ($p<0.01$) with a genotype effect for *Dbp* ($p<0.01$) and *Nr1d1* ($p<0.05$).
