# Supplementary material for: Circadian variation in pulmonary inflammatory responses is independent of rhythmic glucocorticoid signaling in airway epithelial cells
Source: FASEB J. 2018 Jul 2;33(1):126–39. doi: 10.1096/fj.201800026RR (PMC6355062; doi:10.1096/fj.201800026RR)
Supplement: Supplementary file 4 [file fj.201800026RR.sf4.pdf]

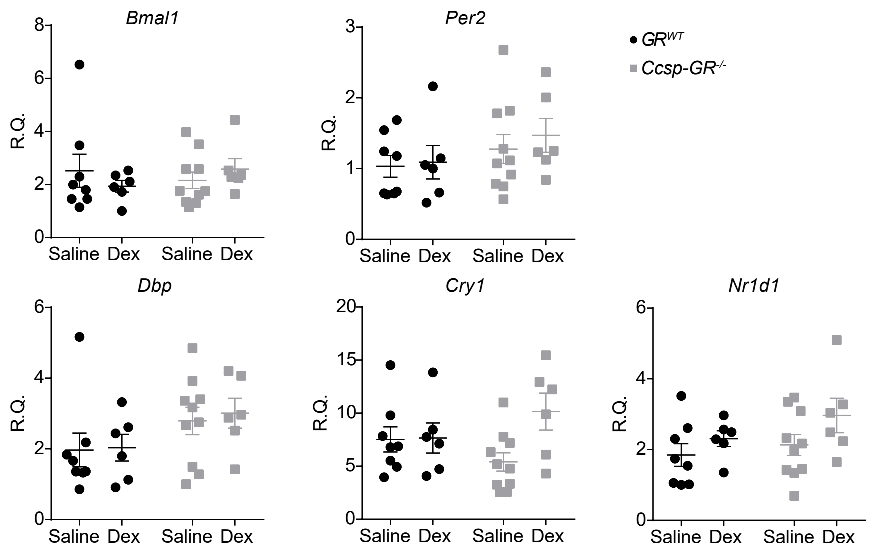

Supplemental Figure 4: Clock gene expression in whole lung after dexamethasone pre-treatment and LPS challenge.

GR^{WT} and *$Ccsp\text{-}GR^{-/-}$* mice were exposed to nebulized LPS at CT0 and culled five hours later. Intraperitoneal injection of either dexamethasone (1mg/kg) or saline vehicle took place 1hr prior to LPS exposure. Whole lung was harvested and analysed for expression of core clock genes. Data were analysed using two-way ANOVA with Sidak's multiple comparisons test, no significant differences were found. *Dex – dexamethasone.*

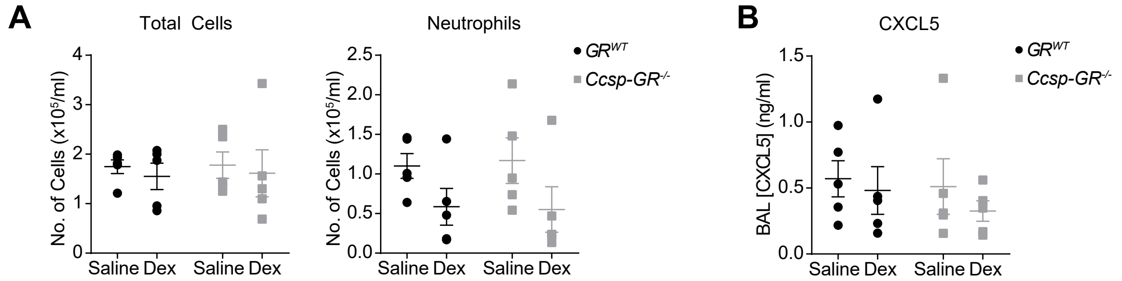

Supplemental Figure 5: Effects of dexamethasone pre-treatment at CT12.

GR^{WT} and *$Ccsp\text{-}GR^{-/-}$* mice were exposed to nebulized LPS at CT12 and culled five hours later. Intraperitoneal injection of either dexamethasone (1mg/kg) or saline vehicle took place 1hr prior to LPS exposure. (A) Quantification of total cells (left) and neutrophils (right) in BAL fluid. (B) Quantification of CXCL5 in BAL fluid. For all panels, n=4-5 and data were analysed using two-way ANOVA followed by Sidak's multiple comparisons test for effects within genotype. No significant differences were found. *Dex – dexamethasone.*
