# Supplementary material for: Circadian variation in pulmonary inflammatory responses is independent of rhythmic glucocorticoid signaling in airway epithelial cells
Source: FASEB J. 2018 Jul 2;33(1):126–39. doi: 10.1096/fj.201800026RR (PMC6355062; doi:10.1096/fj.201800026RR)
Supplement: Supplementary file 5 [file fj.201800026RR.sf5.pdf]

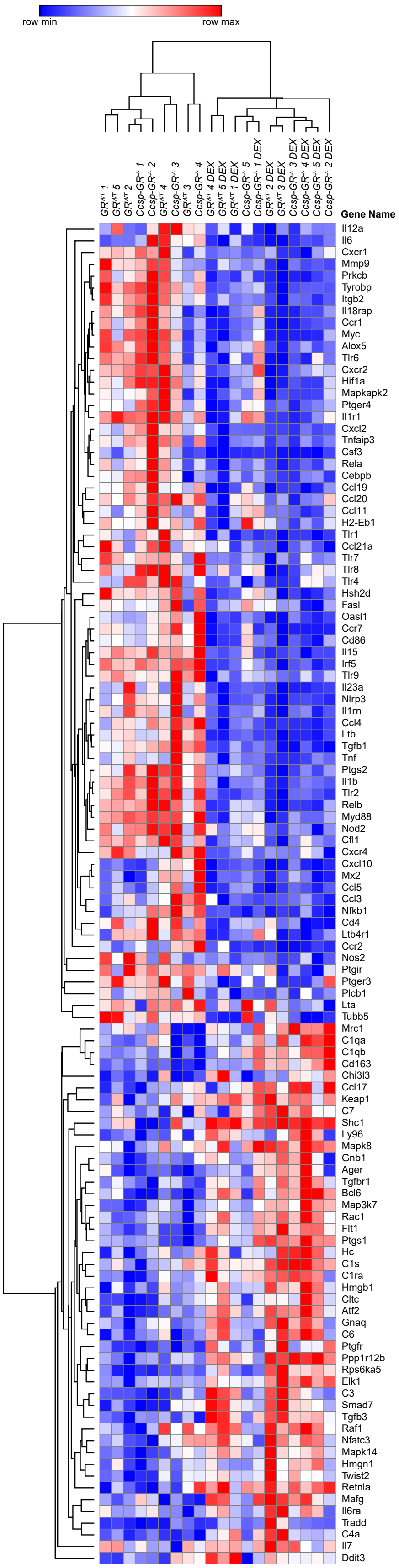

Supplemental Figure 6: Comparison of dexamethasone-responsive genes in *GR*^{WT} and *Ccsp-GR*$^{-/-}$ lung after LPS. Hierarchical clustering of dexamethasone-responsive genes from Nanostring analysis (see Supplemental Table 1 for list of all genes analysed).
