# Supplementary material for: Circadian variation in pulmonary inflammatory responses is independent of rhythmic glucocorticoid signaling in airway epithelial cells
Source: FASEB J. 2018 Jul 2;33(1):126–39. doi: 10.1096/fj.201800026RR (PMC6355062; doi:10.1096/fj.201800026RR)
Supplement: Supplementary file 6 [file fj.201800026RR.sf6.pdf]

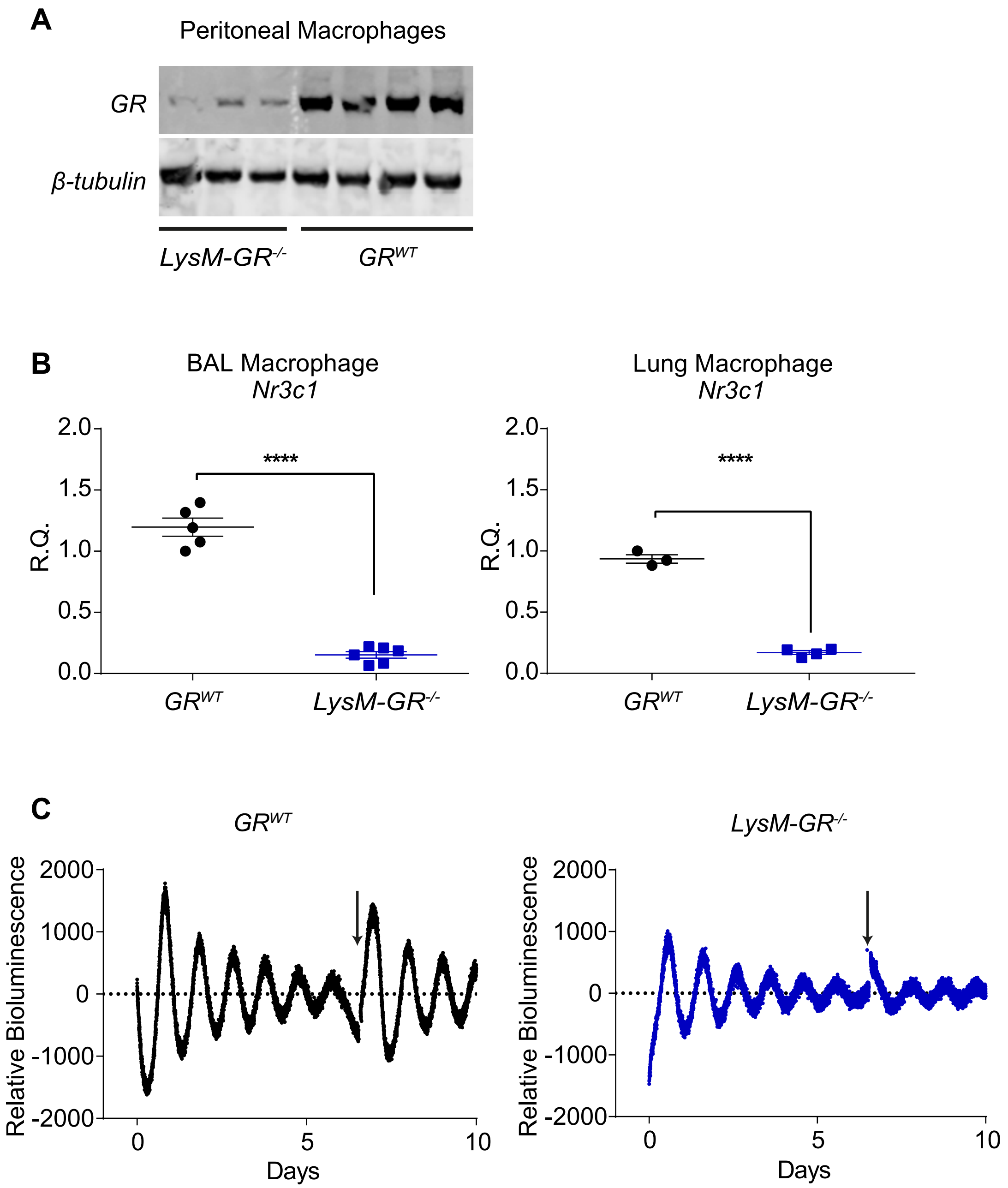

Supplemental Figure 7: Disruption of GR expression and function in *LysM-GR$^{-/-}$* mice. (A) Western blot of protein lysate from peritoneal exudate cells (macrophages) from *LysM-GR$^{-/-}$* mice (left, n=3) and *GR^{WT}* littermate controls (right, n=4). (B) Relative expression of GR mRNA (*Nr3c1*) in sorted macrophages from bronchoalveolar lavage (left) and digested lung (right). Analysis was performed using two-tailed t-test, **** denotes significance at $p<0.0001$. (C) Photon counts over multiple days from *GR^{WT}* (left) and *LysM-GR$^{-/-}$* (right) isolated peritoneal exudate cells. Dexamethasone (Dex) was administered at the indicated times. Plot is representative of 2 independent replicates.
