# Supplementary material for: Circadian variation in pulmonary inflammatory responses is independent of rhythmic glucocorticoid signaling in airway epithelial cells
Source: FASEB J. 2018 Jul 2;33(1):126–39. doi: 10.1096/fj.201800026RR (PMC6355062; doi:10.1096/fj.201800026RR)
Supplement: Supplementary file 7 [file fj.201800026RR.st1.docx]

| **Gene** | **Genotype** | **Treatment** | **Interaction** |  | **Gene** | **Genotype** | **Treatment** | **Interaction** |
| --- | --- | --- | --- | --- | --- | --- | --- | --- |
| Cxcl5 | ** | ns | ns |  | Keap1 | ns | * | ns |
| Il15 | ns | **** | ns |  | Alox5 | ns | * | ns |
| Irf5 | ns | **** | ns |  | Ccl5 | ns | * | ns |
| Tgfb3 | ns | **** | ns |  | Chi3l3 | ns | * | ns |
| Bcl6 | ns | **** | ns |  | C1qa | ns | * | ns |
| Ccr7 | ns | **** | ns |  | Fasl | ns | * | ns |
| Ccl19 | ns | **** | ns |  | Ccr2 | ns | * | ns |
| C3 | ns | **** | ns |  | Cxcr1 | ns | * | ns |
| Atf2 | ns | **** | ns |  | Il6 | ns | * | ns |
| Flt1 | ns | *** | ns |  | Ccl11 | ns | * | ns |
| Myd88 | ns | *** | ns |  | Gnb1 | ns | * | ns |
| Relb | ns | *** | ns |  | Cxcl10 | ns | * | ns |
| Cd86 | ns | *** | ns |  | Plcb1 | ns | * | ns |
| Il1b | ns | *** | ns |  | Ccl3 | ns | * | ns |
| Tyrobp | ns | *** | ns |  | Tlr6 | ns | * | ns |
| C4a | ns | *** | ns |  | Tradd | ns | * | ns |
| Ccl4 | ns | *** | ns |  | Mapk8 | ns | * | ns |
| Prkcb | ns | *** | ns |  | Il6ra | ns | * | ns |
| Ltb | ns | *** | ns |  | Ccl17 | ns | * | ns |
| Tlr2 | ns | *** | ns |  | Ly96 | ns | * | ns |
| Il1r1 | ns | *** | ns |  | Cebpb | ns | * | ns |
| Ptgs1 | ns | *** | ns |  | Hmgn1 | ns | * | ns |
| Tgfb1 | ns | *** | ns |  | Raf1 | ns | * | ns |
| Rac1 | ns | *** | ns |  | Mapkapk2 | ns | * | ns |
| Tubb5 | ns | *** | ns |  | Lta | ns | * | ns |
| Ppp1r12b | ns | *** | ns |  | Hc | ns | * | ns |
| Shc1 | ns | *** | ns |  | Il7 | ns | * | ns |
| Itgb2 | ns | *** | ns |  | Ptgir | ns | * | ns |
| Mafg | ns | *** | ns |  | Tlr4 | ns | * | ns |
| Rps6ka5 | ns | *** | ns |  | Nfatc3 | ns | * | ns |
| Oasl1 | ns | *** | ns |  | C7 | ns | * | ns |
| Mrc1 | ns | *** | ns |  | Mx2 | ns | * | ns |
| Tlr9 | ns | *** | ns |  | Alox12 | ns | ns | ns |
| Nlrp3 | ns | *** | ns |  | Daxx | ns | ns | ns |
| Tlr1 | ns | *** | ns |  | Mef2d | ns | ns | ns |
| Elk1 | ns | *** | ns |  | Gpr44 | ns | ns | ns |
| Cxcl2 | ns | *** | ns |  | NEG_B | ns | ns | ns |
| Ccl20 | ns | *** | ns |  | Ccl2 | ns | ns | ns |
| H2-Eb1 | ns | *** | ns |  | Cxcl9 | ns | ns | ns |
| Nod2 | ns | *** | ns |  | Maff | ns | ns | ns |
| Hsh2d | ns | *** | ns |  | Oas2 | ns | ns | ns |
| Ptgs2 | ns | *** | ns |  | Tnfsf14 | ns | ns | ns |
| Hif1a | ns | *** | ns |  | Ifi27l2a | ns | ns | ns |
| Cd163 | ns | *** | ns |  | Il1rap | ns | ns | ns |
| Map3k7 | ns | *** | ns |  | Mef2c_Mm | ns | ns | ns |
| Tnfaip3 | ns | *** | ns |  | Tslp | ns | ns | ns |
| Cfl1 | ns | *** | ns |  | Areg | ns | ns | ns |
| Tgfbr1 | ns | ** | ns |  | Cd40lg | ns | ns | ns |
| Mapk14 | ns | ** | ns |  | Irf1 | ns | ns | ns |
| Smad7 | ns | ** | ns |  | Fos | ns | ns | ns |
| Ptger4 | ns | ** | ns |  | Creb1 | ns | ns | ns |
| Cxcr4 | ns | ** | ns |  | Mknk1 | ns | ns | ns |
| Cltc | ns | ** | ns |  | Hdac4 | ns | ns | ns |
| Il1rn | ns | ** | ns |  | Hprt | ns | ns | ns |
| Cd4 | ns | ** | ns |  | Rapgef2 | ns | ns | ns |
| C1qb | ns | ** | ns |  | POS_D | ns | ns | ns |
| Tlr7 | ns | ** | ns |  | Cxcl1 | ns | ns | ns |
| Ccr1 | ns | ** | ns |  | Nox1 | ns | ns | ns |
| Ptger3 | ns | ** | ns |  | POS_A | ns | ns | ns |
| Il23a | ns | ** | ns |  | Pla2g4a | ns | ns | ns |
| Mmp9 | ns | ** | ns |  | Tollip | ns | ns | ns |
| Myc | ns | ** | ns |  | POS_C | ns | ns | ns |
| Tnf | ns | ** | ns |  | POS_B | ns | ns | ns |
| C1s | ns | ** | ns |  | Mmp3 | ns | ns | ns |
| C1ra | ns | ** | ns |  | Csf1 | ns | ns | ns |
| Ccl21a | ns | ** | ns |  | Ifit2 | ns | ns | ns |
| Gnaq | ns | ** | ns |  | Cd40 | ns | ns | ns |
| Cxcr2 | ns | ** | ns |  | Nfe2l2 | ns | ns | ns |
| Ptgfr | ns | ** | ns |  | Mx1 | ns | ns | ns |
| Nfkb1 | ns | ** | ns |  | Gnas | ns | ns | ns |
| Ager | ns | ** | ns |  | NEG_C | ns | ns | ns |
| Ddit3 | ns | ** | ns |  | Rhoa | ns | ns | ns |
| Tlr8 | ns | ** | ns |  | C2 | ns | ns | ns |
| Rela | ns | ** | ns |  | Il22ra2 | ns | ns | ns |
| Nos2 | ns | ** | ns |  | Map2k6 | ns | ns | ns |
| C6 | ns | ** | ns |  | Prkca | ns | ns | ns |
| Il12a | ns | ** | ns |  | Il23r | ns | ns | ns |
| Il18rap | ns | * | ns |  | Hspb2 | ns | ns | ns |
| Csf3 | ns | * | ns |  | Jun | ns | ns | ns |
| Retnla | ns | * | ns |  | Masp1 | ns | ns | ns |
| Hmgb1 | ns | * | ns |  | Traf2 | ns | ns | ns |
| Twist2 | ns | * | ns |  | Il18 | ns | ns | ns |
| Ltb4r1 | ns | * | ns |  | Gusb | ns | ns | ns |
|  |  |  |  |  |  |  |  |  |
| **Gene** | **Genotype** | **Treatment** | **Interaction** |  | **Gene** | **Genotype** | **Treatment** | **Interaction** |
| Tlr5 | ns | ns | ns |  | Myl2 | ns | ns | ns |
| POS_F | ns | ns | ns |  | POS_E | ns | ns | ns |
| Oas1a | ns | ns | ns |  | Mapk3 | ns | ns | ns |
| Ptger1 | ns | ns | ns |  | Ltb4r2 | ns | ns | ns |
| Csf2 | ns | ns | ns |  | C9 | ns | ns | ns |
| Mapk1 | ns | ns | ns |  | Max | ns | ns | ns |
| Il22 | ns | ns | ns |  | Mef2b | ns | ns | ns |
| Ccl8 | ns | ns | ns |  | Stat1 | ns | ns | ns |
| Nr3c1 | ns | ns | ns |  | Cd55 | ns | ns | ns |
| Trem2 | ns | ns | ns |  | Grb2 | ns | ns | ns |
| Gapdh | ns | ns | ns |  | Map3k9 | ns | ns | ns |
| Il4 | ns | ns | ns |  | Alox15 | ns | ns | ns |
| Tgfb2 | ns | ns | ns |  | Stat3 | ns | ns | ns |
| Ptk2 | ns | ns | ns |  | Il5 | ns | ns | ns |
| Pik3c2g | ns | ns | ns |  | NEG_F | ns | ns | ns |
| C3ar1 | ns | ns | ns |  | Tlr3 | ns | ns | ns |
| Fxyd2 | ns | ns | ns |  | Cfb | ns | ns | ns |
| Mapkapk5 | ns | ns | ns |  | Ifng | ns | ns | ns |
| Irf3 | ns | ns | ns |  | Ifit1 | ns | ns | ns |
| H2-Ea-ps | ns | ns | ns |  | Ripk2 | ns | ns | ns |
| Iigp1 | ns | ns | ns |  | Mbl2 | ns | ns | ns |
| Ifnb1 | ns | ns | ns |  | Il13 | ns | ns | ns |
| Map2k1 | ns | ns | ns |  | Il1a | ns | ns | ns |
| C8a | ns | ns | ns |  | Stat2 | ns | ns | ns |
| Ccr4 | ns | ns | ns |  | Pdgfa | ns | ns | ns |
| Map3k5 | ns | ns | ns |  | Il2 | ns | ns | ns |
| Ccl7 | ns | ns | ns |  | Tcf4 | ns | ns | ns |
| Cysltr2 | ns | ns | ns |  | Gngt1 | ns | ns | ns |
| Hras1 | ns | ns | ns |  | Cfd | ns | ns | ns |
| Irf7 | ns | ns | ns |  | Il10 | ns | ns | ns |
| Il10rb | ns | ns | ns |  | Ifna1 | ns | ns | ns |
| Il12b | ns | ns | ns |  | Il9 | ns | ns | ns |
| Masp2 | ns | ns | ns |  | Cdc42 | ns | ns | ns |
| Bcl2l1 | ns | ns | ns |  | NEG_A | ns | ns | ns |
| Ccl22 | ns | ns | ns |  | NEG_E | ns | ns | ns |
| Ifit3 | ns | ns | ns |  | Mef2a | ns | ns | ns |
| Map3k1 | ns | ns | ns |  | Nod1 | ns | ns | ns |
| Il17a | ns | ns | ns |  | Cxcl3 | ns | ns | ns |
| Ptger2 | ns | ns | ns |  | Ccl24 | ns | ns | ns |
| Crp | ns | ns | ns |  | Pgk1 | ns | ns | ns |
| Il21 | ns | ns | ns |  | Il11 | ns | ns | ns |
| Birc2 | ns | ns | ns |  | Hmgb2 | ns | ns | ns |
| Ripk1 | ns | ns | ns |  | Kng1 | ns | ns | ns |
| Tbxa2r | ns | ns | ns |  | Limk1 | ns | ns | ns |
| Arg1 | ns | ns | ns |  | C8b | ns | ns | ns |
| Rock2 | ns | ns | ns |  | NEG_G | ns | ns | ns |
| NEG_D | ns | ns | ns |  | NEG_H | ns | ns | ns |
| Defa-rs1 | ns | ns | ns |  | Cysltr1 | ns | ns | ns |
| Mafk | ns | ns | ns |  | Il3 | ns | ns | ns |
| Ccr3 | ns | ns | ns |  | Ifi44 | ns | ns | ns |
| Hspb1 | ns | ns | ns |  | Map2k4 | ns | ns | ns |

**Supplemental Table 1: Effects of dexamethasone treatment before LPS challenge in *Ccsp-GR^-/-^* and *GR^WT^* littermate controls**

Summary of nanostring analysis of whole lung after nebulized LPS challenge at CT0, with either dexamethasone or saline pre-treatment. * denotes significance at p<0.05, ** denotes significance at p<0.01, *** at p<0.001, and **** at p<0.0001.
